# Individual characteristics and student’s engagement in scientific research: a cross-sectional study

**DOI:** 10.1186/1472-6920-12-95

**Published:** 2012-10-15

**Authors:** Ana Salgueira, Patrício Costa, Mónica Gonçalves, Eunice Magalhães, Manuel João Costa

**Affiliations:** 1 Life and Health Sciences Research Institute (ICVS), School of Health Sciences, University of Minho, Campus e Gualtar, 4710-057, Braga, Portugal; 2 Instituto Universitário de Lisboa (ISCTE-IUL), Cis-UL, Avenida das Forças Armadas, 1649-026, Lisbon, Portugal

## Abstract

**Background:**

In light of the increasing recognition of the importance of physician scientists, and given the association between undergraduate research experiences with future scientific activity, it is important to identify and understand variables related to undergraduate students’ decision to engage in scientific research activities. The present study assessed the influence of individual characteristics, including personality traits and socio-demographic characteristics, on voluntary engagement in scientific research of undergraduate medical students.

**Methods:**

For this study, all undergraduate students and alumni of the School of Health Sciences in Minho, Portugal were invited to participate in a survey about voluntary engagement in scientific research activities. Data were available on socio-demographic, personality and university admission variables, as part of an ongoing longitudinal study. A regression model was used to compare (1) engaged with (2) not engaged students. A classification and regression tree model was used to compare students engaged in (3) elective curricular research (4) and extra-curricular research.

**Results:**

A total of 466 students (88%) answered the survey. A complete set of data was available for 435 students (83%).

Higher scores in admission grade point average and the personality dimensions of “openness to experience” and “conscientiousness” increased chances of engagement. Higher “extraversion” scores had the opposite effect. Male undergraduate students were two times more likely than females to engage in curricular elective scientific research and were also more likely to engage in extra-curricular research activities.

**Conclusions:**

This study demonstrated that student’ grade point average and individual characteristics, like gender, openness and consciousness have a unique and statistically significant contribution to students’ involvement in undergraduate scientific research activities.

## Background

Advances in medical diagnosis and therapeutics walk hand in hand with scientific development in other disciplines like biochemistry, pharmacology or physics, as future medical care depends on today’s scientific research [1,2]. More and more, physicians are called to assume a central role in the scientific research/patient care partnership. They are increasingly expected to communicate with researchers and convey clinical and translational research findings to patients and to the general public. Moreover, they are required to contribute actively to the pursuit of new knowledge, bringing clinical needs into research and taking research findings into clinical practice [3,4].

However, available data point to a decrease in the numbers of physician-scientists[5-7]. Amongst the reasons for such decline are less financial incentives, a large emphasis on clinical practice during undergraduate medical training, and insufficient or inadequate exposure to research prior to the choice of a professional pathway [8-10].

The reasons why and when physicians choose careers in academic medicine have been explored and evidence has been found connecting graduate and postgraduate research [11]: (a) participating in research methodology courses and more positive attitudes towards science and scientific research in medicine [12,13]; (b) participating in required research experiences and publishing research reports [14,15] or participating in postgraduate research [16]; (c) engaging in intensive research experiences and receiving a faculty appointment with research responsibility [17] and (d) publishing research as an undergraduate medical student and/or pursuing an MD/PhD and choosing academic medicine [5,18-20]. Evidence also shows that engagement in undergraduate extra-curricular scientific activity results in a higher rate of publication after graduation [21].

Medical schools can provide undergraduate students with opportunities to engage in research and thus have an important role in nurturing the interests and in developing the research skills of future physicians. Previous studies show that limitations in time, lack of mentors, insufficient training in research methodologies, and a perception that the undergraduate student’s research work is not properly recognized are amongst the factors that discourage medical students from pursuing undergraduate research activities [9,22].

Research in Higher Education has revealed that undergraduates’ career choices, attitudes, values, and future behaviors are deeply influenced by what students do during college [23,24] and that individual variables, like gender or parental education, are associated with undergraduate students’ engagement in extra-curricular activities [23,25]. Also, personality traits have been shown to have predictive validity regarding outcomes like behavior [26], academic performance in medical students [27-29] and medical specialty choice [30-32]. Surprisingly, the influence of undergraduate medical students’ individual characteristics on their involvement in research activities has not deserved attention. Current literature on student engagement in scientific research focuses on programs and how they can contribute to the pipeline for physician scientists. Identifying the individual variables that mediate this behavior is important to understand how engagement in research can be enhanced.

Our aim in this study was to assess the influence of individual characteristics, including personality traits and socio-demographic characteristics, on voluntary engagement in scientific research of undergraduate medical students.

## Methods

### Institutional context

The study took place in the School of Health Sciences at the University of Minho, Portugal (ECS/UM). Having a built-in research institute, the school explicitly emphasizes to students the importance of research and offers them opportunities to engage by: i) promoting research-related activities within the curriculum, ii) challenging students to engage in scientific activities during curricular electives and iii) providing opportunities for extracurricular research activities.

The independent variables in this study - personality, socio-demographic factors and University admission grade point average (GPA) - are available from the start of the medical school (2001) as part of an ongoing longitudinal study in which this research project was included. The Portuguese Data Protection Authority approved the longitudinal study. Participation in the longitudinal study is voluntary, confidential and written informed consent is asked, of all participants, every time a new piece of data is collected, and is to be integrated in the study. All data is anonymised before analysis.

### Variables, instruments and data collection procedures

#### Independent variables

Personality measurements were obtained with the Portuguese version of NEO-FFI (NEO Five-Factor Inventory). NEO-FFI is a shortened version of the NEO PI-R [33,34] and measures 5 dimensions of personality (openness, conscientiousness, extraversion, agreeableness, and neuroticism) using a 5 point likert scale (from 0 - strongly disagree to 4 - strongly agree) with 12 items for each dimension. Scores for each dimension range from 0 to 48. The Portuguese version of NEO-FFI [35] includes 60 items with Cronbach’s Alpha ranging from 0.71 (Openness) to 0.81 (Conscientiousness) and corroborates the well-established reliability, factorial structure, and cross-cultural communalities of personality according to gender, age, and educational differences. The surveys on socio-demographic variables (gender, age, parents’ education background – 1st or 2nd generation student) and University admission data (choosing the ECS/UM as the 1st option, GPA - scores ranging from 0–200 used to rank students for university access) were custom-made by the research team. To measure the number of opportunities each student had to participate in undergraduate research, we created a variable called “total of opportunities” corresponding to the number of years the student was in the school, until the time of this study. These surveys are collected annually at the beginning of every academic year for each new cohort, either online or on paper.

#### Dependent variables

We asked students if they had ever been involved in undergraduate scientific research activities. All the research activities covered by the survey implied a choice made by the student to engage in scientific research either (1) as part of their curriculum (during elective curricular areas units that take place every year and allow the students to choose between research, clinical rotations, or social/humanitarian work), or (2) as an extra-curricular activity such as (2.1) summer laboratory rotations as part of the application requirements for the MD-PhD program, (2.2) one full year part-time research scholarships for students or (2.3) on their own venture. Research type, frequency, and duration of participation were not taken into consideration in this study. Students were categorized into two groups: (i) unengaged students with no involvement in undergraduate scientific research activities and (ii) engaged students with involvement in undergraduate scientific research activities, either extra-curricular or elective, at least once (if they had at least one positive answer for any of the types of participation). Additionally, we divided all the “engaged students” into two groups according to the type of undergraduate scientific research activities: elective curricular (CA) or extra-curricular (ECA). As the two groups are not mutually exclusive (some students engaged curricular research activities, as well as extra-curricular), all the students with at least one extra-curricular research activity were included in the second group. Self-reported information in the participation survey was manually verified by matching the respondents’ answers with the school’s official records of participation.

The participation questionnaire was administered online at the conclusion of the 2009/2010 academic year.

### Sample exclusion criteria

Besides the normal process for university admission, students can get into medical school using special access processes for athletes, military, islands and immigrants. These students’ GPA is lower. All the students with GPA lower than 179.8 (the lowest GPA for the normal admission process since 2001) were discarded from the analysis (n = 106; GPA: M = 163.29; SD = 11.15).

We also excluded students who only developed scientific activities during the compulsory master’s thesis (required for graduation) (n = 60).

### Statistical analysis

To test the representativeness of our sample, we compared all the independent variables for the “respondent” and “non respondent” students in the research activities questionnaire using a Student *t*-test (for continuous variables) and the *χ*^2^ test (for categorical variables).

Subjects with complete sets of information for all independent variables were selected for the following statistical analyses. Data were analyzed using IBM SPSS Statistics version 19. We performed a binary logistic regression model to test which student characteristics could explain engagement in undergraduate scientific research activities. The analysis was performed using the backward LR method (at each step, the variables in the model were analyzed to remove those that do not significantly contribute to the model). The model was obtained in 3 steps. For internal validation of results, a bootstrap analysis with 1000 samples was performed using the Enter method for the step 3 model. We used a “Classification and Regression Tree” model to explore the differences between two groups of engaged students: (1) those who chose to engage in undergraduate scientific research activities during their elective curricula areas and (2) those who decided to engage in undergraduate scientific research activities as an extra-curricular activity. This is a non-parametrical approach used to explain responses on a categorical dependent variable that can be used as an exploratory technique instead of the more traditional methods. It also has an advantage over regression in its ability to detect nonlinear relationships. For this model we used CRT as the growing method, pruning on misclassification error (1 SE rule) and Gini measure for goodness of fit (impurity criteria). The minimum number of isolates in a parent node was set to 10 and 5 for the child nodes. The independent variable “opportunities” was included in the model as the “influence variable”.

## Results

### Sample

**Figure 1:**
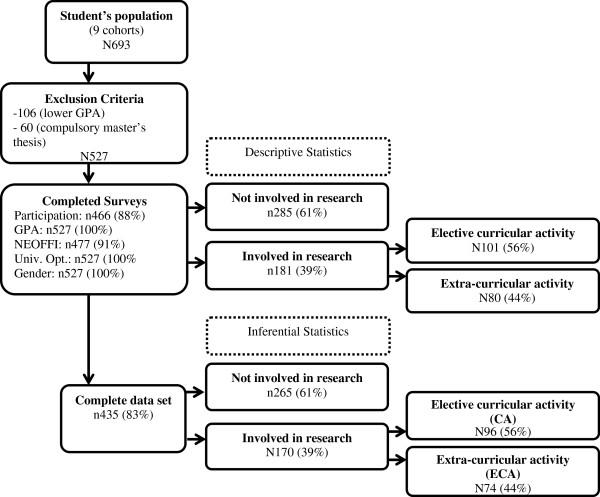
Sample.

We surveyed all students and alumni from ECS/UM (9 cohorts) on their participation in scientific research activities during medical school (n = 693). After applying the exclusion criteria, the final target population consisted of 527 students. A total of 466 (88%) students completed the online survey about participation in scientific research activities. Participation rates varied between the 9 cohorts from 72% to 92% (cohort1 92%; cohort2 90%; cohort3 92%; cohort4 91%; cohort5 91%; cohort6 92%; cohort7 92%; cohort8 72%; cohort9 91%). As for the other longitudinal study surveys, 527 students provided information for GPA, 477 for personality, 527 for university option, and 527 for gender. Figure 1 illustrates the attrition from the original number of students to the sample.

A complete set of data (personality, GPA, and socio-demographic variables) was available for 435 of the 527 eligible students (83%). 364 (69%) were females and age was quite homogeneous (M = 18.28; SD = 1.22). GPA for our sample ranged from 179.8 to 196.3 (M = 186.20; SD = 3.30).

### Cross-validation of self-reported information and sample validation

Mismatch between students’ self-report and official records was less than 2%. Comparison between “respondents” and “non respondents” showed no statistically significant differences for each one of the independent variables (see Table 1).

**Table 1:** Sample validation: comparison between “respondents” and “non respondents” for each independent variable

**INDEPENDENT VARIABLES**		**Non respondents**		**Respondents**		**Total**		**Mean difference**
		**n = 62**		**n = 465**		**n = 527**		**(T-Test/ *χ***^**2**^**Test)**
		**n (%)**	**Mean/SD**	**n (%)**	**Mean/SD**	**n (%)**	**Mean/SD**	
Opportunities		62 (100%)	3.6/1.9	465 (100%)	3.4/1.9	527 (100%)	3.4/1.9	t(525) = .946, n.s.
GPA		62 (100%)	186/3.2	465 (100%)	186.1/3.3	527 (100%)	186.1/3.3	t(525) = −.404, n.s.
Neuroticism		41 (66%)	24.7/6.8	437 (94%)	23.9/7.7	478 (91%)	23.9/7.6	t(476) = .804, n.s.
Extroversion		41 (66%)	32.6/7.9	437 (94%)	31.2/5.4	478 (91%)	31.3/5.7	t(475) = 1.663, n.s.
Openness		41 (66%)	29.5/5.3	436 (94%)	30.5/5.4	477 (91%)	30.4/5.4	t(475) = 1.373, n.s.
Agreeableness		41 (66%)	33.7/5.6	437 (94%)	33.9/5.3	478 (91%)	33.9/5.3	t(476) = −.160, n.s.
Conscientiousness		n41 (66%)	32.2/5.8	n436 (94%)	33.7/6.6	n477 (91%)	33.6/6.6	t(475) = 1.209, n.s.
Gender	F	43 (69%)	--	321 (69%)	--	364 (69%)	--	*χ*^2^(1, N = 527) = 0.02, n.s.
	M	19 (31%)	--	144 (31%)	--	163 (31%)	--	
This university was my first option		42 (68%)	--	356 (77%)	--	--398 (76%)		*χ*^2^(1, N = 398) = 2.576, n.s.

### Research Engagement

Over more than half (61%) of the participants had never engaged in undergraduate scientific research activities. Within the groups of students with involvement in undergraduate scientific research activities (N = 181) 56% engaged in an elective curricular activity and 44% in an extra-curricular activity.

### Students’ characteristics associated with engagement in research

The variables in the regression model significantly predicted engagement in undergraduate scientific research activities (G2(8) = 123.220; p < .001). Results show that male students are two times more likely to participate than females. For every five points increment in GPA, students increase their probability of participation by 67% (1.67 times more likely). Five more points in openness increase the chance of participation by 57% (1.57 times more likely) and in conscientiousness by 26% (1.26 times more likely). Scoring five points higher for extraversion decreases the chances of participation by 33% (0.67 times less likely). For every additional year in medical school there is a 1.6 fold increase in the likelihood of participation. No statistical significance was found for neuroticism nor agreeableness.

Using a cut point value of 0.5, the model correctly classifies 74% of the subjects (62% of participants and 81% of non-participants), 13% more than chance. Overall, the model explains 33% of the dependent variable’s observed variance (Nagelkerke Pseudo-R^2^ = .334). Hosmer-Lemeshow test showed a good model fit (*χ*^2^HL(8) = 10.378, p = .239). The odds ratios for the original regression model and the bootstrap model are shown in Table 2. In the bootstrap analysis, the small bias and standard error values, the fact that all B values are inside the confidence intervals and the fact that statistical significance for all variables is maintained, confirm the stability of the model.

**Table 2:** Odds ratios for the regression model: original and bootstrap

**INDEPENDENT VARIABLES**	**Step 1**		**Step 2**		**Step 3**								
	**Model**		**Model**		**Model**				**Bootstrap (1000 samples)**				
	**B**	**Exp(B)**	**B**	**Exp(B)**	**B**	***χ***^**2**^_**WALD**_	**Exp(B)**	Exp (B*5)	**Bias**	**Std. Error**	**Sig. (2-tailed)**	**Conf. Int. (95%)**	
OPPORTUNITIES	.480	1.616***	.480	1.616***	.475	48.860***	1.608***	--	.007	.068	.001	.358	.623
**PERSONALITY TRAITS**													
Extroversion	-.080	.923**	-.080	.923**	-.080	10,490**	.923**	0.670	-.003	.027	.004	-.138	-.032
Neuroticism	-.030	.971	-.030	.970	-.030	2,875	.971	--	-.002	.017	.059	-.062	.004
Openness to experience	.090	1.094***	.089	1.093***	.090	15,141***	1.094***	1.567	.001	.025	.001	.046	.146
Conscientiousness	.046	1.047*	.046	1.048*	.047	6,126*	1.049*	1.268	.001	.019	.005	.011	.088
Agreeableness	-.042	.959	-.042	.959	-.044	3,647	.957	--	-.002	.025	.064	-.096	.003
**SOCIO-DEMOGRAPHICS**													
Gender	.700	2.014**	.700	2.014**	.707	7,376**	2.029**	--	.014	.262	.004	.214	1.251
1st Generation Student	-.043	.958	--	--	--		--	--	--	--	--	--	--
**ADMISSION DATA**													
GPA	.095	1.099*	.094	1.098*	.103	8,051**	1.108**	1.672	.005	.039	.008	.030	.179
University choice	.175	1.191	.176	1.193	--		--	--	--	--	--	--	--
Constant	−19.794	.000	−19.627	.000	−21.123	9,943	.000	--	-.801	7.107	--	−35.421	−7.579
N	435		435		435								
**Pseudo R-square**	**.335**		**.335**		**.334**								
−2 log likelihood	458.544		458.577		458.903								

*p < .05; **p < .01; ***p < .001.

**Figure 2:**
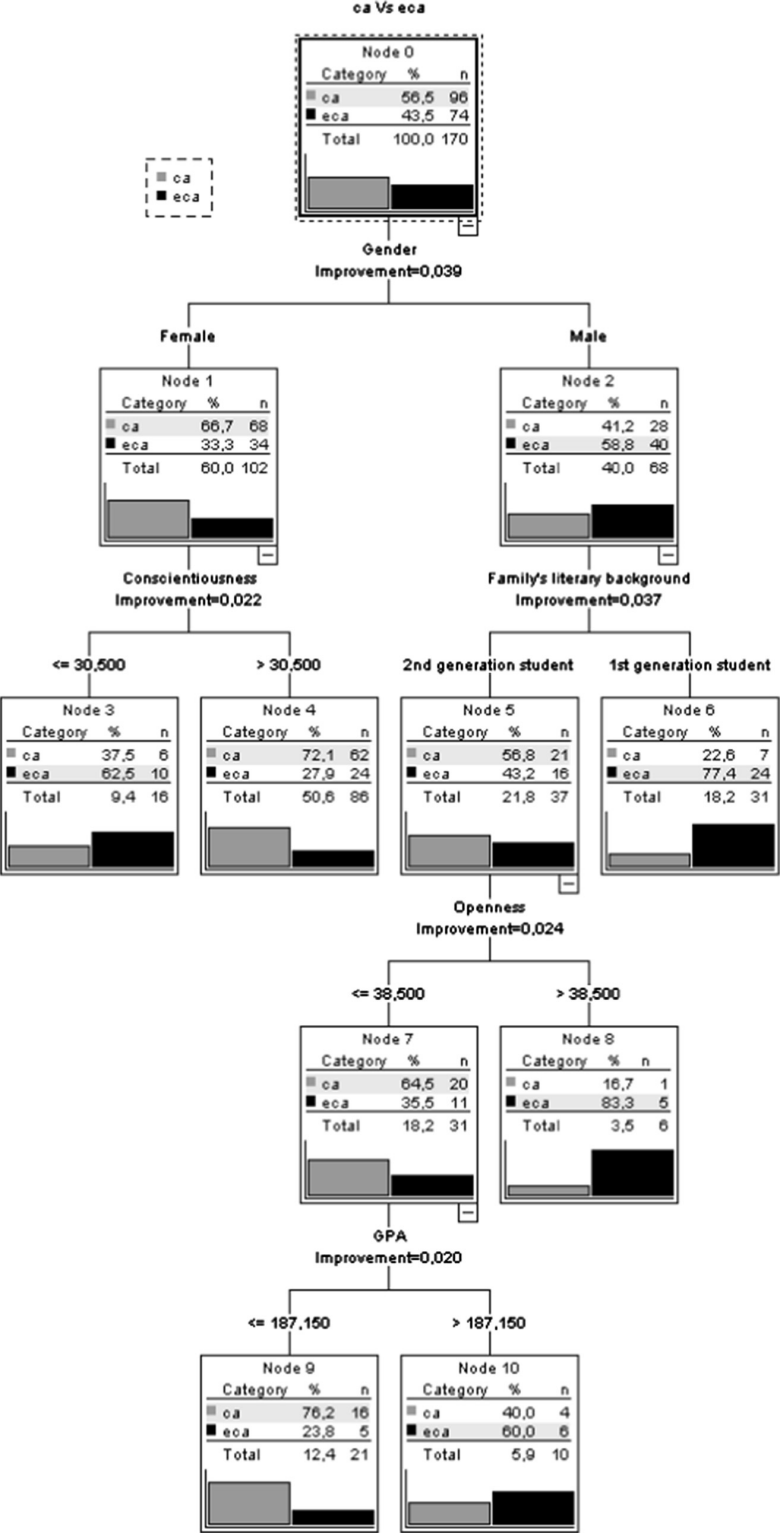
CRT model: decision tree.

A “Decision Tree” (Figure 2) was used to identify the variables that discriminate between the students engaged in “Extra-curricular” undergraduate scientific research activities (ECA) (n = 74) and those engaged in “Curricular” undergraduate scientific research activities (CA) (n = 96). The final tree consisted of 10 nodes, 6 of which were terminal nodes.

The CRT method automatically excluded agreeableness, neuroticism, extroversion, and university option, as these variables did not make a statistically significant contribution to the final model. The first split was based on student gender. The proportion of male students involved in ECA was higher. First generation male students are more involved in ECA than second generation ones. Second generation males with higher levels of openness and higher GPA tend to be more involved in ECA. Female participation in ECA is related to lower levels of conscientiousness.

The risk estimate for the “Decision Tree” was .29 (SE.035). Overall the model correctly classified 72% of the subjects (81% CA and 61% ECA).

## Discussion

Collectively, our results show that three out of the Big Five dimensions of personality (openness to experience, conscientiousness, and extraversion), gender, and GPA have a unique and statistically significant contribution to students’ involvement in undergraduate scientific research activities.

To the best of our knowledge this is the first study to consider the contribution of student’s individual characteristics to engagement in undergraduate scientific research activities. Also, this study takes into consideration a student’s actual research participation behavior, rather than future intentions of participation or positive attitudes towards research and science [12,13].

Although the associations observed were statistically significant, they were modest, which is not surprising given the complexity of human behavior. That is, other individual and contextual factors might influence student’s engagement (e.g. students’ autonomy levels or availability of role models amongst the faculty). In fact, previous studies determined that personality variables usually account for about 14% of the variance in behavior [26]. Our model, by adding other individual characteristics to personality traits, explained 33% of the variance, thus adding an important dimension to the understanding of complex decision-making behaviors.

Personality predicts behavior to the extent that it can influence the psychological state of an individual and predispose him to action. Considering that “open individuals” are characterized as being intellectually curious, creative, and more adaptable to novel situations, their higher involvement is congruent with the type of work and intellectual curiosity demanded by scientific research. Motivation, persistence, careful planning, and the ability to delay gratification are important traits for this activity and are common to individuals with high conscientiousness scores; thus, it is not surprising that both openness and conscientiousness positively influence students’ participation. In contrast, “extroverted individuals” tend to value more socially stimulating activities and are less likely to concentrate on demanding cognitive tasks, which is likely to explain a smaller involvement of highly extroverted individuals [36].

Higher GPA was linked to greater engagement in research. One of the reasons underlying this relation might be that students with higher GPAs could be more confident in their ability to use their transferable skills (for example, communication skills and time management) to tackle the demands that come with scientific research participation.

Also, results showed that male students are more likely to be involved in research. Gender imbalances in engagement have been reported [22] and might be caused by cultural and social factors that keep women from participating (for example, lower levels of autonomy and unavailability of female role models) or by different self-perceptions of competence between males and females. In fact, a study by Burgoyne et al. [37] demonstrated that male students felt significantly more competent in transferable and research-specific skills and biological statistics. It is also possible that female students are more focused on academic performance and prefer to invest their time and efforts in what they perceive to be more curriculum-related activities. Interestingly, the categorization of two sub-samples according to the type of involvement (elective curricular or extra-curricular), revealed the proportion of women engaged in scientific research in extra-curricular settings was even lower. However, this proportion increased if we only considered the female students with lower “conscientiousness” scores, suggesting that female students might be more focused on curricular performance.

Besides finding the effect of individual characteristics on undergraduate scientific research activities engagement, we found that some of these dimensions (gender, conscientiousness, openness, and GPA) are also related to the type of extra-curricular involvement students choose, which further strengthens our findings. Interestingly, parents’ education was also a factor that influenced student engagement in extra-curricular undergraduate scientific research activities. In fact, for males, being a “first generation student” seems to have an impact on the type of involvement they choose to have. Available data from other studies points in different directions: first generation students were found to have lower educational aspirations and to be less involved in campus activities [38]. However, these studies were not done with medical students and it is quite possible that the very demanding selection process for medical school admission might be selecting first generation students for whom their family’s educational background is not relevant for their educational attainment. Also, changes in the Portuguese educational, social, and economic reality in the past two decades might mean new career opportunities for first generation students, encouraging them, and their families, to invest in different activities that can contribute to their professional success.

If one assumes that student engagement in research is a positive behavior that should be encouraged, taking student characteristics into consideration might result in more targeted efforts of recruitment and hold greater promise in contributing to the sustainability of the physician-scientist career pipeline.

### Limitations

Caution must be used in making generalizations from the study results in light of the following limitations. Although the participants in our study were exposed to similar curricula, faculty, staff, and educational opportunities (all of which can be discarded as confounding factors in the present study), they all originated from one single institution. Even though we considered the number of opportunities the students had to engage in research, the fact that not all of the students were in the same curricular stage is a limitation. Bootstrap analysis supports the validity of our regression model, but further confirmation in prospective studies and with future cohorts of students is needed to further address the issue. Because the number of students engaging in research activities is low, our CRT sample was small. For that reason, no cross-validation method was used and we allowed small minimum numbers of subjects in the child nodes. Further analysis with greater samples is crucial. Future studies that take into account these shortcomings will certainly contribute to a better definition and characterization of the best predictors of engagement in research activities. Our study discards all variables related to institutional context and it also does not explore subsequent behavior of engagement exhibited by the students (e.g. abandoning research after they have engaged versus maintaining the behavior in a consistent manner). Future qualitative research might give an insight on other important variables associated with student’s engagement in scientific research.

## Conclusions

Our results showed that male students are two times more likely to participate in research activities than females. Students with higher GPA and higher scores of openness and conscientiousness are also more likely to engage in research activities. On the contrary, higher scores in extraversion decrease the likelihood of participation. Other personality dimensions like neuroticism and agreeableness have no predictive power over students’ engagement in research.

Our findings also add some insight on student’s characteristics related to student’s participation in extracurricular research activities, showing that male, 1st generation students are more involved and that female participation in ECA is related to lower levels of conscientiousness.

## Competing interests

The authors declare that they have no competing interests.

## Authors’ contributions

All authors designed the study. AS, MG and EM administered the surveys. AS and PC developed the statistical analysis. AS wrote the first draft of the manuscript. All authors have reviewed and approved the text of the manuscript.

## Funding/Support

The longitudinal study was sponsored by a grant from the Portuguese Foundation for Science and Technology - project PTDC/ESC/65116/2006.

## Pre-publication history

The pre-publication history for this paper can be accessed here:

http://www.biomedcentral.com/1472-6920/12/95/prepub
